# *Candida albicans* Chitin Increases Arginase-1 Activity in Human Macrophages, with an Impact on Macrophage Antimicrobial Functions

**DOI:** 10.1128/mBio.01820-16

**Published:** 2017-01-24

**Authors:** Jeanette Wagener, Donna M. MacCallum, Gordon D. Brown, Neil A. R. Gow

**Affiliations:** MRC Centre for Medical Mycology, Aberdeen Fungal Group, School of Medicine, Medical Sciences & Nutrition, Institute of Medical Sciences, University of Aberdeen, Aberdeen, United Kingdom; Washington University School of Medicine

## Abstract

The opportunistic human fungal pathogen *Candida albicans* can cause a variety of diseases, ranging from superficial mucosal infections to life-threatening systemic infections. Phagocytic cells of the innate immune response, such as neutrophils and macrophages, are important first-line responders to an infection and generate reactive oxygen and nitrogen species as part of their protective antimicrobial response. During an infection, host cells generate nitric oxide through the enzyme inducible nitric oxide synthase (iNOS) to kill the invading pathogen. Inside the phagocyte, iNOS competes with the enzyme arginase-1 for a common substrate, the amino acid l-arginine. Several pathogenic species, including bacteria and parasitic protozoans, actively modulate the production of nitric oxide by inducing their own arginases or the host’s arginase activity to prevent the conversion of l-arginine to nitric oxide. We report here that *C. albicans* blocks nitric oxide production in human-monocyte-derived macrophages by induction of host arginase activity. We further determined that purified chitin (a fungal cell wall polysaccharide) and increased chitin exposure at the fungal cell wall surface induces this host arginase activity. Blocking the *C. albicans*-induced arginase activity with the arginase-specific substrate inhibitor *N*ω-hydroxy-nor-arginine (nor-NOHA) or the chitinase inhibitor bisdionin F restored nitric oxide production and increased the efficiency of fungal killing. Moreover, we determined that *C. albicans* influences macrophage polarization from a classically activated phenotype toward an alternatively activated phenotype, thereby reducing antimicrobial functions and mediating fungal survival. Therefore, *C. albicans* modulates l-arginine metabolism in macrophages during an infection, potentiating its own survival.

## IMPORTANCE

The availability and metabolism of amino acids are increasingly recognized as crucial regulators of immune functions. In acute infections, the conversion of the “conditionally essential” amino acid l-arginine by the inducible nitric oxide synthase to nitric oxide is a resistance factor that is produced by the host to fight pathogens. Manipulation of these host defense mechanisms by the pathogen can be key to successful host invasion. We show here that the human opportunistic fungal pathogen *Candida albicans* influences l-arginine availability for nitric oxide production by induction of the substrate-competing host enzyme arginase-1. This led to a reduced production of nitric oxide and, moreover, reduced eradication of the fungus by human macrophages. We demonstrate that blocking of host arginase-1 activity restored nitric oxide production and increased the killing potential of macrophages. These results highlight the therapeutic potential of l-arginine metabolism in fungal diseases.

## INTRODUCTION

*Candida albicans* is an opportunistic fungal pathogen that frequently colonizes the mucosal surfaces of healthy individuals without causing infection (1). However, in individuals in whom the normally suppressive endogenous bacterial microflora is disturbed, in patients experiencing severe trauma or surgery, immunocompromised individuals, or those undergoing immunosuppressive therapies, *C. albicans* is a frequent cause of mucocutaneous or disseminated infections (2). Phagocytic cells, such as macrophages and neutrophils, are important mediators of innate immunity and are responsible for developing a robust antimicrobial response after recognition and ingestion of pathogens (3). The synthesis of antimicrobial effectors, such as reactive oxygen species (ROS) and reactive nitrogen species (RNS), is an important cornerstone of the phagocyte antimicrobial response. Nitric oxide (NO) in macrophages is produced by the inducible nitric oxide synthase (iNOS, NOS2), which catalyzes the conversion of the amino acid l-arginine to NO and citrulline. NO is a central component of phagocyte innate immunity and can react with superoxide to peroxynitrite, an effective cytotoxic antimicrobial agent against intracellular and extracellular pathogens, such as *Mycobacterium tuberculosis* and *Escherichia coli* (4).

iNOS is not present in resting cells but can be induced by immunostimulatory type 1 cytokines, such as interferon gamma (IFN-γ), tumor necrosis factor alpha (TNF-α), and interleukin-1 (IL-1), but also by microbial cell wall components, such as lipopolysaccharide (LPS) and lipoteichoic acid (LTA), during an infection (5). The availability of l-arginine is a rate-limiting factor in NO synthesis (6), and in mammalian cells, iNOS competes with the enzyme arginase-1 (Arg-1) for the substrate l-arginine. Arginase-1 can be induced in macrophages by type 2 cytokines, such as IL-4, IL-13, IL-10, and transforming growth factor beta (TGF-β), inhibiting iNOS functions and leading to increased humoral immunity, tissue repair, and allergic responses (7). Additionally, polyamines produced in the arginase pathway downregulate proinflammatory cytokine release. The critical interplay between arginase-1 and iNOS is important in influencing the outcome of an infection, and several pathogens have been shown to regulate this important pathway either by modulating l-arginine availability through induction of host arginases or by using their own arginases to metabolize host l-arginine (reviewed in reference 4).

*C. albicans* has been shown to actively block the production of NO by macrophages, although the modulating factor and the underlying mechanisms are not known (8–10). Here, we link *C. albicans* NO suppression to cell wall changes occurring during the *C. albicans* response to macrophages and adaptation to the phagosome environment. We show that the fungal cell wall polysaccharide chitin induces host arginase-1 expression and activity, thereby suppressing NO synthesis. Moreover, this interference with host l-arginine metabolism shifts classically activated macrophages toward an alternative activated phenotype, enhancing *C. albicans* survival.

## RESULTS

### *C. albicans* influences host arginase activity.

**FIG 1:**
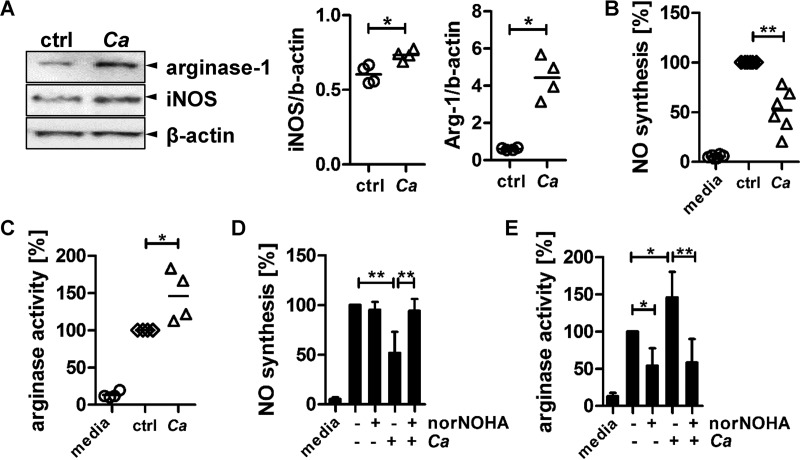
*C. albicans* impacts macrophage NO production by inducing host arginase activity. (A) iNOS and arginase-1 protein levels in IFN-γ/LPS-activated human-monocyte-derived macrophages after coincubation with live *C. albicans* (*Ca*) for 3 h (*n =* 4; *, *P* < 0.05); (B) NO inhibition by *C. albicans* as determined by measuring nitrite levels (*n =* 6; **, *P* < 0.01); (C) arginase enzyme activity as determined by measuring the conversion of arginine to urea (*n =* 4; *, *P* < 0.05); (D and E) NO synthesis (D) and arginase activity (E) of IFN-γ/LPS-activated macrophages left untreated or preincubated with 25 µM nor-NOHA for 30 min to block arginase activity before coincubation with live *C. albicans* cells for 3 h (*n =* 6; *, *P* < 0.05; **, *P* < 0.01). Arginase activity and NO levels are expressed as percentages of levels generated by IFN-γ/LPS-activated macrophages alone (control [ctrl]). All data are presented as mean values ± SD.

We hypothesized that *C. albicans* promotes its own survival in phagocytes by manipulating the availability of l-arginine for NO synthesis. We therefore analyzed the induction of iNOS and arginase-1 protein expression in IFN-γ- and LPS-activated human monocyte-derived macrophages, cocultured with *C. albicans* (multiplicity of infection [MOI] = 1) for 3 h by immunoblotting (Fig. 1A). *C. albicans* significantly increased both iNOS and arginase-1 (Arg-1) protein expression, although the total increase in protein level was higher for Arg-1 (7.43-fold ± 1.61-fold) than for iNOS (1.21-fold ± 0.05-fold). We next examined NO production by measuring the amount of nitrite in the supernatants of the same cocultures (Fig. 1B). Interestingly, although a slightly increased iNOS protein level was detected, we observed a 50% reduction of NO released from the activated macrophages in the presence of *C. albicans* (Fig. 1B). However, the observed increased arginase-1 protein expression led to a 40% increase in arginase activity in the analyzed cell lysates (Fig. 1C).

To prove that the reduced NO production was due to the observed host arginase-1 induction and not due to upregulation of the fungal NO detoxification mechanism, we added the selective arginase-1 inhibitor *N*ω-hydroxy-nor-arginine (nor-NOHA) to block arginase activity (11). This restored *C. albicans*-suppressed NO levels in cocultured macrophages to levels observed in macrophages that were not exposed to *C. albicans* (Fig. 1D). The increased arginase activity in macrophages cocultured with *C. albicans* was also reduced by nor-NOHA to levels observed in nor-NOHA-treated activated macrophages in the absence of *C. albicans* (Fig. 1E). Therefore, we conclude that the presence of *C. albicans* increased arginase-1 activity in IFN-γ- and LPS-activated human monocyte-derived macrophages, limiting the availability of l-arginine for NO production by iNOS.

### Increased arginase activity is chitin mediated and leads to reduced macrophage antimicrobial function.

**FIG 2:**
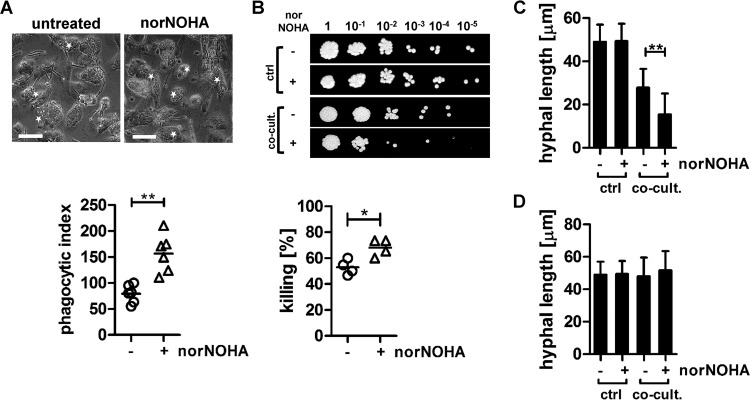
The inhibition of arginase activity impacts macrophage functions. IFN-γ/LPS-activated macrophages were either untreated or preincubated with 25 µM nor-NOHA for 30 min to block arginase activity before being coincubated with live *C. albicans* cells for 3 h. (A) The uptake of *C. albicans* by the macrophages was determined microscopically. Pictures shown are representative of three independent experiments. Stars indicate macrophages containing multiple *Candida* cells. Scale bar = 50 µm. The phagocytic index (PI) was calculated according to the following formula: PI = (percentage of phagocytic cells containing ≥1 *Candida* cell) × (mean number of *Candida* cells/phagocytic cell containing *Candida*) (*n =* 6; **, *P* < 0.01). A minimum of 100 macrophages was counted for each condition. (B) *C. albicans* survival in control culture and macrophage cocultures (co-cult.) was determined by CFU microdilution spot assays. CFU are expressed as percentages of *C. albicans* cells killed by macrophages compared to numbers present in control samples (*C. albicans* alone) (*n =* 4; *, *P* < 0.05). The spot assay shown is representative of three independent experiments. (C and D) Hypha formation of controls and phagocytosed (C) and nonphagocytosed (D) *C. albicans* cells was determined microscopically, and hyphal length was measured using ImageJ. A minimum of 100 hyphae/germ tube was counted for each condition (*n =* 4; **, *P* < 0.01). All data are presented as mean values ± SD.

Next, we determined the impact of increased arginase activity and blocked NO production on antimicrobial macrophage function and *C. albicans* germ tube formation. Blocking arginase activity with nor-NOHA significantly increased fungal uptake (Fig. 2A) and killing (Fig. 2B) by IFN-γ- and LPS-activated human monocyte-derived macrophages. Moreover, reduced arginase activity led to decreased *C. albicans* hyphal extension inside macrophage phagosomes (Fig. 2C). Germ tube formation of nonphagocytosed *C. albicans* was not affected by nor-NOHA treatment (Fig. 2D). Incubation of *C. albicans* with nor-NOHA in the absence of macrophages did not impact *Candida* viability (Fig. 2B) or germ tube formation (Fig. 2C and D).

**FIG 3:**
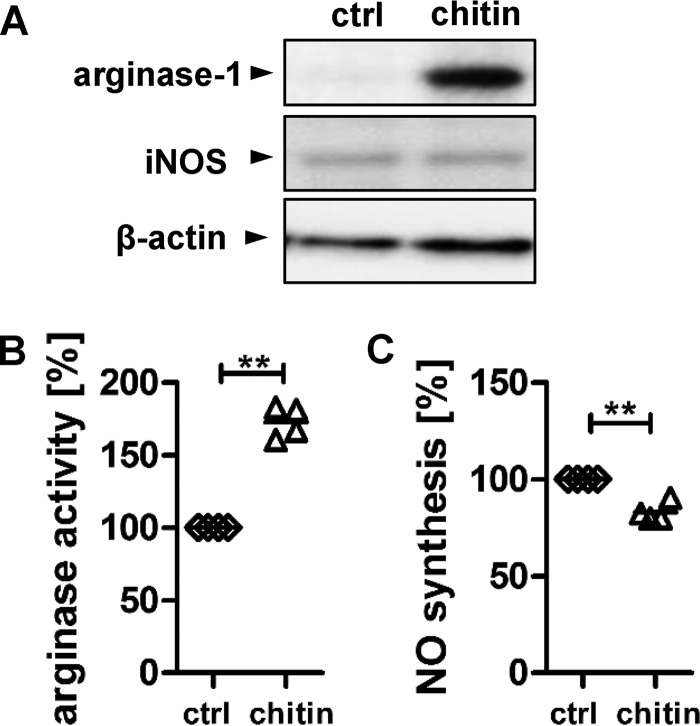
Purified fungal chitin induces arginase activity. (A) iNOS and arginase-1 protein levels in IFN-γ/LPS-activated macrophages stimulated with 10 µg/ml purified *C. albicans* chitin for 3 h. Blots shown are representative of two independent experiments with four donors. (B and C) Arginase enzyme activity induced by purified *C. albicans* chitin (B) and NO inhibition by *C. albicans* chitin (C). Arginase activity and NO levels are expressed as percentages of levels generated by IFN-γ/LPS-activated macrophages alone (ctrl) (*n =* 4; **, *P* < 0.01). All data are presented as mean values ± SD.

**FIG 4:**
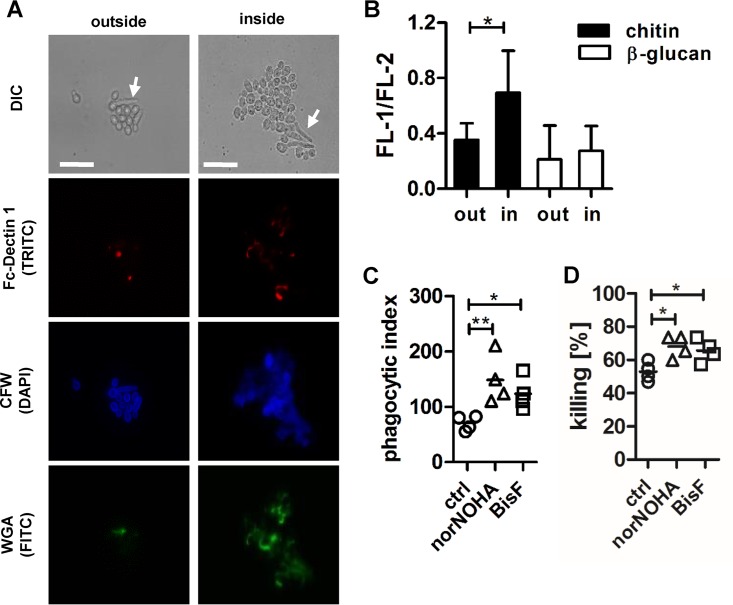
Increased chitin exposure of phagocytosed *C. albicans*. (A) *C. albicans* was cocultured with IFN-γ/LPS-activated macrophages for 3 h. Nonphagocytosed *C. albicans* cells were collected from the supernatants and washes of adherent macrophages. Phagocytosed *C. albicans* cells were released by macrophage lysis. Fungal cells were stained for β-glucan and chitin exposure using Fc–dectin-1–tetramethyl rhodamine isocyanate (TRITC) (red), wheat-germ agglutinin (WGA)-FITC (green), and calcofluor white (blue). Pictures shown are representative of results from three independent experiments. (B) The fluorescence (FL) intensity of Fc–dectin-1–TRITC, WGA-FITC, and CFW staining was determined using ImageJ. Minimally 50 cells were analyzed. β-glucan and chitin exposure is expressed as FL-1 (WGA-FITC or Fc–dectin-1–TRITC)/FL-2 (CFW) (**, *P* < 0.01). (C and D) IFN-γ/LPS-activated macrophages were left untreated or were preincubated with 25 µM nor-NOHA or 50 µM bisdionin F (BisF) for 30 min before being coincubated with live *C. albicans* cells for 3 h. (C) The uptake of *C. albicans* cells by the macrophages was determined microscopically, and minimally 100 macrophages were counted for each condition to determine the PI (*n =* 4; **, *P* < 0.01). (D) *C. albicans* survival in macrophage cocultures was determined by a CFU microdilution assay. CFU are expressed as percentages of *C. albicans* cells killed by macrophages compared to those present in control samples (*C. albicans* alone) (*n =* 4; *, *P* < 0.05). All data are presented as mean values ± SD.

Arginase-1 induction in macrophages can be induced by type 2 cytokines, such as IL-4, IL-10, and IL-13 (7). Chitin is a cell wall polysaccharide which is a potent stimulator of allergic responses, and we showed previously that purified fungal chitin induces IL-10 secretion in human myeloid cells (12). Therefore, we tested the ability of purified fungal chitin to induce arginase protein expression and arginase activity in IFN-γ- and LPS-activated human monocyte-derived macrophages (Fig. 3). *C. albicans*-derived chitin increased arginase-1 protein levels but did not induce iNOS expression (Fig. 3A). Arginase activity was, however, significantly increased in chitin-stimulated macrophages (Fig. 3B), whereas NO synthesis was reduced (Fig. 3C). Next, we determined whether chitin on the surface of *C. albicans* was accessible for immune recognition by macrophages. We stained *C. albicans* cells that had been cocultured with IFN-γ- and LPS-activated human monocyte-derived macrophages for 3 h, both phagocytosed and nonphagocytosed cells, for β-glucan (red) and chitin (green) exposure (Fig. 4A). *C. albicans* cells recovered from macrophage phagosomes showed significantly increased chitin surface exposure, but no major changes in β-glucan exposure were observed (Fig. 4B). To test whether chitin released from phagocytosed *C. albicans* by the activity of acidic mammalian chitinase (AMCase) was responsible for the reduced antimicrobial activity of the macrophages, we preincubated the macrophages with the AMCase-specific inhibitor bisdionin F. Inhibition of AMCase activity resulted in an increase in *C. albicans* phagocytosis (Fig. 4C) and killing (Fig. 4D) by the macrophages, comparable to the increase observed with nor-NOHA treatment (Fig. 4C and D). Neither chemical (nor-NOHA or bisdionin F) altered chitin surface exposure (see Fig. S1A in the supplemental material), *C. albicans* viability (Fig. S1B and Fig. 2B), or hyphal extension (Fig. S1C and Fig. 2C) when added directly to the cells.

10.1128/mBio.01820-16.1 FIG S1  nor-NOHA and bisdionin F do not impact *C. albicans* growth. *C. albicans* was cultured in the presence of 25 µM nor-NOHA or 50 µM bisdionin F for 3 h. (A) Fungal cells were stained for mannan, β-glucan, and chitin exposure using concanavalin A (ConA) (red), Fc–dectin-1–tetramethyl rhodamine isocyanate (TRITC) (purple), wheat-germ agglutinin (WGA)-FITC (green), and calcofluor white (CFW) (blue). Images are representative of two independent experiments. Scale bar = 20 µm. (B) *C. albicans* survival was determined by a CFU microdilution spot assay. The spot assay results shown are representative of two independent experiments. (C) The hyphal formation of *C. albicans* was determined microscopically, and hyphal length was measured using ImageJ. Minimally 20 hyphae/germ tube were counted for each condition. All data are presented as mean values ± SD. DIC, differential interference contrast. Download FIG S1, TIF file, 0.9 MB. Copyright © 2017 Wagener et al. 2017 Wagener et al. This is an open-access article distributed under the terms of the Creative Commons Attribution 4.0 International license.

Therefore, exposed chitin in the cell wall of *C. albicans* led to increased activity of arginase-1 in IFN-γ/LPS-activated human monocyte-derived macrophages, thereby reducing *C. albicans* killing.

### A chitin-mediated increase in arginase activity requires active fungal cell wall remodeling and correlates with *C. albicans* virulence.

**FIG 5:**
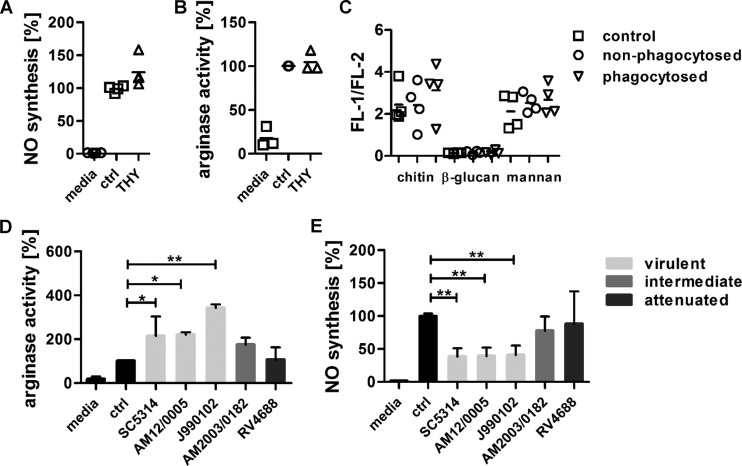
A chitin-mediated increase in arginase activity requires active fungal cell wall remodeling and correlates with *C. albicans* virulence. IFN-γ/LPS-activated macrophages were coincubated with thimerosal-treated *C. albicans* yeast cells (THY) or live *C. albicans* cells for 3 h. (A and E) NO inhibition by THY (A) and live *C. albicans* cells (E) (*n =* 4; **, *P* < 0.01). (B and D) Arginase enzyme activity (*n =* 3; *, *P* < 0.05). Arginase activity and NO levels are expressed as percentages of the levels generated by IFN-γ/LPS-activated macrophages alone (ctrl). (C) THY were cocultured with IFN-γ/LPS-activated macrophages for 3 h. Nonphagocytosed THY were collected from the supernatants and washes of adherent macrophages. Phagocytosed THY were released by macrophage lysis. Fungal cells were stained for cell wall mannans (concanavalin A), β-glucan (Fc–dectin-1–TRITC), and chitin (WGA-FITC and calcofluor white). Fluorescence intensity of staining was determined using ImageJ. Minimally 20 cells were analyzed. Mannan, β-glucan, and chitin exposure is expressed as FL-1 (concanavalin A, WGA-FITC, or Fc–dectin-1–TRITC)/FL-2 (CFW). All data are presented as mean values ± SD.

Increased chitin surface exposure may be driven by *C. albicans* cell wall remodeling in response to the phagosome environment, similar to the increased chitin deposition in the cell wall described after an attack by neutrophils (13) and/or unmasking by the actions of host enzymes or exposure to echinocandins (e.g., caspofungin) (14). We therefore coincubated IFN-γ/LPS-activated human monocyte-derived macrophages with nonviable yeast cells (thimerosal-treated yeast cells [THY]) for 3 h and determined macrophage NO production and arginase-1 activity, as well as the surface exposure of β-glucan and chitin of control samples, for both nonphagocytosed and phagocytosed THY (Fig. 5). Nonviable *C. albicans* cells did not significantly reduce NO production (Fig. 5A) or induce arginase activity (Fig. 5B). Cell wall staining revealed that phagocytosis by macrophages did not significantly alter the cell surface presentation of β-glucan or chitin in nonviable yeast cells during the experimental time course (3 h) (Fig. 5C).

*C. albicans* cell wall remodeling during infection is predicted to have a significant impact on signaling pathways important for mounting a protective immune response (15, 16). *In vivo* infection with different *C. albicans* strains from different multilocus sequence type (MLST) clades revealed that mice deficient in the major β-glucan receptor dectin-1 can exhibit a protective immune response in a strain-dependent manner. Interestingly, the pathogenicity of the strains used in this study correlated with increased chitin levels observed *in vivo* (16). We therefore tested four additional *C. albicans* strains with different virulence attributes for their ability to increase macrophage arginase activity and to reduce NO production (Table 1; Fig. 5). Levels of induction of arginase activity and reduction of NO synthesis varied between the strains and correlated with their reported *in vivo* pathogenicity (Table 1) (17). Strains classified as virulent (SC5314, J990102) in a systemic mouse model of *C. albicans* infection and an untested clinical isolate (AM12/0005) induced the highest arginase activity in IFN-γ/LPS-activated human monocyte-derived macrophages after 3 h and significantly blocked NO production (Fig. 5D and E). The intermediate (AM2003/0182) and attenuated (RV4688) strains did not significantly change arginase activity (Fig. 5D) or NO synthesis (Fig. 5E).

**TABLE 1:** Characteristics of strains used in this study

Strain	Anatomical source	MLST clade	Virulence attribute^a^	Reference
SC5314	Blood	1	Virulent	50
AM2012/0005	Blood	Unknown	Not tested	Unpublished
AM2003/0182	Blood	2	Intermediate	17
J990102	Vagina	3	Virulent	17
RV4688	Blood	4	Attenuated	17

a Mouse systemic model.

We therefore conclude that increased chitin exposure is actively driven by fungal cell wall remodeling and that the levels of arginase activity induction and reduction of NO synthesis are *C. albicans* strain specific and correlate with the virulence attributes of the tested strains.

### Purified fungal chitin and *C. albicans* surface-exposed chitin influence the macrophage phenotype.

**FIG 6:**
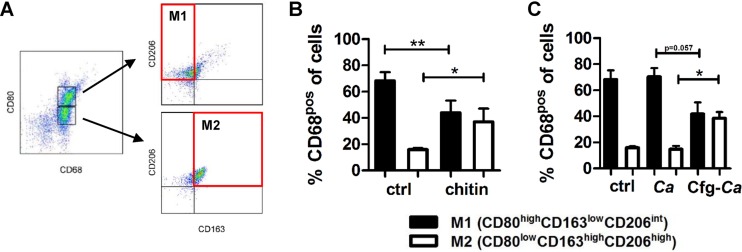
Purified fungal chitin and increased cell wall chitin exposure of *C. albicans* changes macrophage polarization. (A) The gating strategy was that classically activated macrophages (M1) were defined as CD68^pos^ CD80^high^ CD163^low^ CD206^int^ (where “pos” is positive and “int” is intermediate) and alternatively activated macrophages (M2) as CD68^pos^ CD80^low^ CD163^high^ CD206^high^. (B and C) IFN-γ/LPS-activated macrophages were stimulated with 10 µg/ml purified *C. albicans* chitin for 24 h (B) or with heat-inactivated *C. albicans* (with or without caspofungin treatment [Cfg]) (C), and the expression of CD80, CD163, and CD206 was determined by flow cytometry (*n =* 4; *, *P* < 0.05; **, *P* < 0.01). All data are presented as mean values ± SD.

Macrophage activation depends on stimuli present in their immediate environment. Macrophages adapt to environmental signals, such as cytokines produced by surrounding cells (7). This leads to flexibility in macrophage programing, enabling macrophages to switch readily from one functional phenotype to another in response to changing microenvironmental signals. Pathogen- and damage-associated molecular patterns, together with IFN-γ, polarize macrophages toward an M1 phenotype, characterized by the production of ROS and RNS, which facilitate the killing of microbial pathogens. In contrast to M1 macrophages, alternatively activated M2 macrophages suppress inflammation and facilitate wound repair. High arginase activity has been described for the M2 macrophage phenotype, which is important in facilitating the production of prolines required for wound healing (18). To determine whether the chitin-induced increase in arginase activity is also impacted the macrophage phenotype, we analyzed the expression of macrophage phenotype-specific markers by flow cytometry to distinguish macrophage phenotypes (Fig. 6).

Up to 70% of the *in vitro-*differentiated IFN-γ/LPS-activated macrophages expressed markers characteristic of the M1 phenotype (Fig. 6). Stimulation with purified fungal chitin significantly reduced this M1 population while simultaneously significantly increasing the proportion of cells expressing M2 markers (Fig. 6B). To overcome technical restrictions on using live *C. albicans* cells in flow cytometry due to hyphal formation, we mimicked the increased chitin surface expression observed in live *C. albicans* cells (Fig. 4) by pretreating yeast cells with caspofungin, a cell wall-targeting antifungal drug which increases the exposure of both chitin and β-glucan (19, 20), before inactivating the yeast cells chemically with thimerosal to preserve the cell wall changes. Macrophages stimulated with untreated *C. albicans* cells showed no change in polarization markers, whereas macrophages stimulated with caspofungin-treated yeast cells showed a decrease in the M1 population and a significant increase in the M2 macrophage population (Fig. 6C), which was comparable to levels induced by purified fungal chitin alone (Fig. 6B). Therefore, the recognition of purified fungal chitin and exposed cell wall chitin shifted the equilibrium from M1 to M2 macrophages.

### Macrophage phenotype is an important determinant of the antifungal response to *C. albicans.*

**FIG 7:**
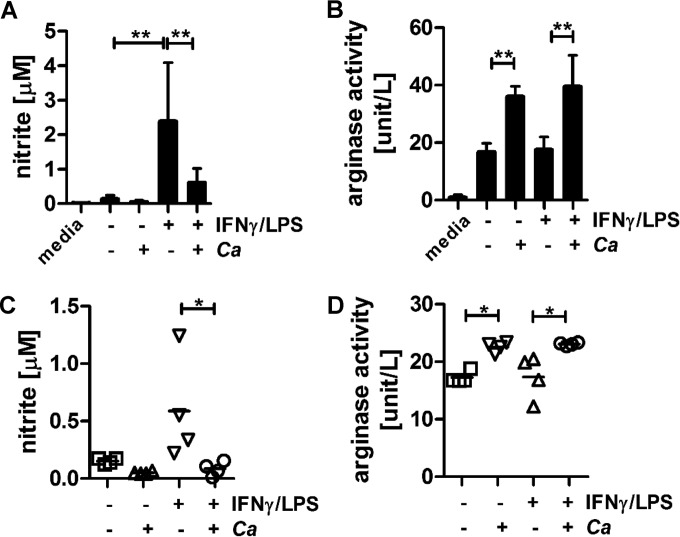
*C. albicans* induced host arginase activity in resting and tissue-resident macrophages. (A and C) NO inhibition by *C. albicans* in human monocyte-derived macrophages (A) and mouse peritoneal macrophages (C) (*n =* 4); (B and D) *C. albicans*-induced arginase enzyme activity in human monocyte-derived macrophages (B) and mouse peritoneal macrophages (D) (*n =* 4). Data are presented as mean values ± SD. *, *P* < 0.05; **, *P* < 0.01.

Immune surveillance and tissue homeostasis is an important task of tissue-resident macrophages. Tissue-resident macrophages, alongside other tissue-resident cells, such as stromal cells, mast cells, and dendritic cells, respond to pathogens by initiating inflammation. The initial pathogen encounter drives the influx of neutrophils and inflammatory monocytes, the source of inflammatory macrophages (M1), as part of the antimicrobial defense (21). Tissue-resident macrophages are classified as M2-like due to their functional similarity to M2 macrophages. We therefore tested the influence of *C. albicans* on arginase activity and NO production in tissue-resident macrophages. We repeated the coincubation experiments with nonactivated (resting) and IFN-γ/LPS-activated human monocyte-derived macrophages (Fig. 7A and B) and, in addition, challenged nonactivated and IFN-γ/LPS-activated murine peritoneal (tissue-resident) macrophages (Fig. 7C and D) with *C. albicans*. We found that resting and tissue-resident macrophages secreted very low levels of nitric oxide (Fig. 7A and C) and that NO production increased upon activation with IFN-γ/LPS (Fig. 7B and D), which supports the results of prior reports (5). Coincubation with *C. albicans* did not impact NO levels in nonactivated macrophages (Fig. 7A and C). However, coincubation with *C. albicans* significantly increased the arginase activity in both nonactivated human macrophages and mouse peritoneal macrophages (Fig. 7B and D).

**FIG 8:**
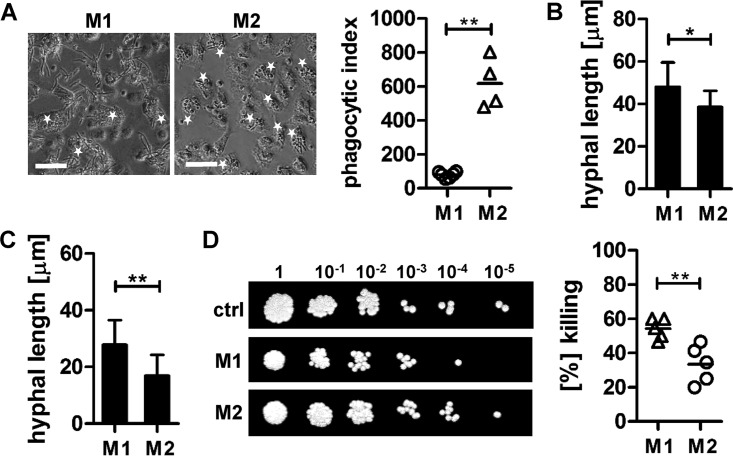
Alternatively activated macrophages restrict *C. albicans* growth but fail to eradicate the fungus. The uptake of *C. albicans* by IFN-γ/LPS (M1)- or IL-4 (M2)-activated macrophages was determined microscopically, and minimally 100 macrophages were counted for each condition. The PI was calculated (*n =* 4; **, *P* < 0.01). Scale bar = 50 µm. (B and C) The hypha formation of nonphagocytosed (B) and phagocytosed (C) *C. albicans* cells was determined microscopically, and hyphal length was measured using ImageJ. Two hundred hyphae/germ tube were counted for each condition (*, *P* < 0.05; **, *P* < 0.01). (D) *C. albicans* survival in control culture and macrophage cocultures was determined by a CFU microdilution spot assay. The spot assay results shown are representative of three independent experiments. CFU are expressed as percentages of *C. albicans* cells killed by macrophages compared to numbers of cells in control samples (*C. albicans* alone) (*n =* 5). All data are presented as mean values ± SD.

Alternative activation of macrophages is complex, and several macrophage subtypes have been described in the literature (7, 18). In general, M2 macrophages are marked by increased anti-inflammatory cytokine production (e.g., IL-10, TGF-β, IL-1RA) and increased expression of C-type lectins (CTLs) (e.g., dectin-1, dendritic cell-specific intercellular adhesion molecule-3-grabbing nonintegrin [DC-SIGN], and mannose receptor) and scavenging receptors for the uptake of cellular debris (7). Alternative activation of macrophages due to Th2 responses (e.g., IL-4 and IL-13) has been shown to promote fungal persistence, allergy, and disease relapse, and neutralization of endogenous IL-4 has beneficial effects on the survival of mice systemically infected with *C. albicans* (22). When we challenged alternatively (IL-4) activated macrophages with live *C. albicans*, we found that these macrophages were more potent in their ability to phagocytose *C. albicans* than IFN-γ/LPS-activated (M1) macrophages (Fig. 8A). Although hypha formation of nonphagocytosed (Fig. 8B) and phagocytosed *C. albicans* (Fig. 8C) cells was significantly suppressed compared to that in M1 macrophages, alternatively activated macrophages showed a markedly reduced ability to kill *C. albicans* (Fig. 8B).

In summary, classical macrophage activation is important for pathogen eradication, and an increased alternative activation of macrophages may result in increased *C. albicans* persistence following tissue invasion.

## DISCUSSION

Amino acid metabolism has been increasingly recognized as an important modulator of immune responses (23). The amino acid l-arginine is classified as “conditionally essential,” depending on the developmental state and health status of an individual (6). During an infection, l-arginine availability has been shown to be a crucial regulator of immune functions, and several pathogens, such as *Salmonella* and *Leishmania* spp., can impact host l-arginine metabolism in various ways (4). In this study, we show that the human-pathogenic fungus *C. albicans* alters macrophage nitric oxide production by affecting host arginase-1 expression and activity. Furthermore, we identified fungal cell wall chitin as an important component that mediates arginase-1 induction in human macrophages.

Previous studies have reported that *C. albicans* can suppress nitric oxide production in IFN-γ/LPS-activated primary mouse macrophages and in activated cell lines (RAW264.7 and J774.A1) (8–10). A common observation in these studies was that NO inhibition was highest when live *C. albicans* cells were in direct contact with macrophages. A decrease in iNOS expression (mRNA/protein) was observed with *C. albicans* (8, 10) and coculture (9) supernatants. In contrast, our results with human IFN-γ/LPS-activated macrophages showed a slight increase in iNOS protein induced by *C. albicans*, but this did not result in higher enzyme activity as measured by nitric oxide production. Therefore, the reduced NO response of human macrophages to *C. albicans* was not likely to be due to transcriptional downregulation of the iNOS gene. Inhibition of NO production was also not mediated by *Candida*-generated competitive inhibitors (9). We determined that reduced NO production was most likely to be due to competition for the l-arginine substrate by the host arginase-1 enzyme. Blocking arginase-1-mediated consumption of its substrate l-arginine by the substrate inhibitor nor-NOHA restored NO production in *C. albicans*-challenged human macrophages and decreased *C. albicans* survival.

Using a transwell system, Collette et al. (9) showed that direct cell-cell contact was not necessary for reduced nitric oxide production and that it also occurred in response to conditioned cell-free supernatants from macrophage-*Candida* cocultures. They described a “secreted” mediator as a small, aqueous, and heat-stable compound. Chitin is a heat-stable carbohydrate that is released from the fungal cell wall by human chitinases during macrophage-*Candida* interactions (12) and is, therefore, a credible candidate for this class of mediator. Chitin is also known to induce alternatively activated macrophages in mouse lungs, which are marked by a high expression of the enzyme arginase-1 (24). Although chitin in the native cell wall is covered by a layer of mannoproteins, we showed that chitin can be exposed during *C. albicans*-macrophage interactions and that *C. albicans* chitin is a strong inducer of arginase-1 activity in human macrophages. Exposure of underlying cell wall layers has also been shown to occur when *C. albicans* is attacked by neutrophils (13). Using a specific inhibitor (bisdionin F) for the human acidic mammalian chitinase (AMCase) to prevent chitin release from the cell walls of phagocytosed *C. albicans* cells, we demonstrated that chitin release negatively influenced macrophage antimicrobial functions. Moreover, chitin recognition influenced macrophage polarization, pushing macrophages from a classically activated, antimicrobial phenotype toward an alternatively activated phenotype, thereby decreasing antimicrobial activity. The ability of *C. albicans* to influence macrophage activation and phenotype has been show previously (25, 26), although the impact on antimicrobial functions and *C. albicans* survival was not determined.

Alternative activation of macrophages by *C. albicans* in the context of the local immune response may have either beneficial or detrimental effects for the host. The activation of a protective Th1/Th17 response is important for the host to clear an infection, but uncontrolled inflammation leads to overinduction and immunopathology. Maintaining the correct balance of the host’s immune response is therefore crucial for the clearance of an infection and the resolution of inflammation. In this context, the predominant, but not exclusive, induction of classical macrophages has been shown to be protective against several fungal species, including *Cryptococcus neoformans*, *Aspergillus fumigatus*, and *C. albicans*, whereas a predominant induction of alternative macrophages is implicated in the protection against *Pneumocystis* spp. (18). Interestingly, the recognition of chitin and the activity of the mammalian chitinase chitotriosidase have recently been shown to mediate pathological Th2-associated inflammation during *C. neoformans* lung infection *in vivo* (27). Further, the abrogation of IL-4 receptor α-dependent alternative activation of macrophages can mediate resistance to pulmonary *C. neoformans* infection marked by reduced arginase-1 expression and increased nitric oxide production (28). The importance of developing a protective Th1/Th17 response over a nonprotective Th2 response in clearing fungal infections has been described previously (29), and the negative impact of IL-4 in macrophage activation during fungal infection is linked to fungal persistence (22).

We show here that alternatively (IL-4) activated human macrophages have increased phagocytic capabilities and an enhanced ability to suppress *C. albicans* hypha formation, but they fail to eradicate the fungus. *C. albicans* can utilize amino acids, such as l-arginine, as an alternative carbon source under conditions where other carbon sources, such as glucose, are limited (30). Such conditions are predicted to exist inside phagosomes (31, 32), and gene transcription analysis confirmed that expression of *C. albicans* genes coding for arginine import and degradation are increased after phagocytosis by macrophages (30). Moreover, increased arginine catabolism was shown to be important for phagosome alkalinization, hyphal induction, and macrophage escape (30, 33). The restricted availability of host l-arginine as an energy source, due to high arginase-1 activity in alternatively activated macrophages, can explain the reduced hypha formation (intra- and extracellular) observed in these experiments.

In humans, arginase-1 is constitutively expressed in granulocytes and has been shown to participate in neutrophil antifungal activity, possibly through arginine depletion (34, 35). On the other hand, arginase-1 release after granulocyte cell death at the site of inflammation and the exocytosis of arginase-1-containing granules due to “frustrated phagocytosis” has been shown to suppress T-cell proliferation and cytokine synthesis (34, 36, 37). Sustained arginase-1 activity can also deplete extracellular l-arginine levels, thereby impairing not only T-cell function (38, 39) but also NK cell activation (40). Increased arginase activity occurs as a result of tissue injury and subsequent wound healing, cancer, cystic fibrosis, or sepsis, thereby increasing susceptibility to secondary infections, including mycoses (41–44). Alternatively, increased macrophage arginase-1 expression has been shown to be important in the suppression of T-cell-mediated immunopathology in a mouse model of *Mycobacterium tuberculosis* infection (45). These observations highlight the importance of l-arginine metabolism mediated by arginase-1 in the resolution of inflammation.

In summary, our findings demonstrate that changes in the exposure of *C. albicans* cell wall chitin can influence macrophage functions by impacting host l-arginine metabolism. Our data reveal an increased fungal killing potential of macrophages if either host chitinase (bisdionin F) or arginase (nor-NOHA) activity is reduced. Therapeutic inhibition of arginase-1 has been shown to have beneficial effects for other diseases, such as asthma, cancer, and parasitic infections, and shows potential as a therapeutic target to explore in fungal diseases.

## MATERIALS AND METHODS

### Monocyte isolation, purification, and macrophage differentiation.

Blood from healthy volunteers was collected according to the local guidelines and regulations, approved by the College Ethics Review Board of the University of Aberdeen (CERB/2012/11/676). Peripheral blood mononuclear cells (PBMCs) were isolated by Ficoll-Paque Plus (GE Healthcare) density centrifugation according to the manufacturer’s instructions. Further, highly pure unlabeled monocytes were obtained from PBMCs by depletion of nonmonocytes using a magnetically activated cell sorting (MACS) system together with a human pan-monocyte isolation kit (Miltenyi Biotec, Inc.). Purified monocytes were washed twice with phosphate-buffered saline (PBS), and cells were suspended in RPMI 1640 (Dutch modification) supplemented with 10% heat-inactivated fetal bovine serum (FBS), 2 mM l-glutamine, 1 mM sodium pyruvate, 1% minimum essential medium (MEM), nonessential amino acids (NEAA [100×]; Gibco), 100 U/ml penicillin, and 100 mg/ml streptomycin. An aliquot of 50 ng/ml recombinant human granulocyte-macrophage colony-stimulating factor (hGM-CSF) (Gibco) was added to differentiate monocytes into an M1-like polarized phenotype, and recombinant hM-CSF (Gibco) was added to generate an M2-like polarized phenotype. Cells were seeded into cell culture dishes at a density of 1 × 10^6^ cells/cm^2^ and incubated at 37°C in a humidified atmosphere containing 5% CO_2_, and fresh medium containing recombinant cytokines was added every other day. After 5 to 7 days, cells were collected, seeded into 12- or 24-well plates at a density of 5 × 10^5^ cells or 2 × 10^5^ cells/well, respectively, and left overnight to adhere. M1-polarized macrophages were fully activated by adding 100 ng/ml IFN-γ and LPS, and M2-polarized macrophages were activated by adding 20 ng/ml IL-4 for 24 h.

### Isolation of mouse peritoneal macrophages.

C57BL/6 (wild-type) mice were bred and housed under pathogen-free conditions in the registered animal facility at the University of Aberdeen. All animal work at the University of Aberdeen is regulated under the UK Home Office’s Animals (Scientific Procedures) Act of 1986 (ASPA) (46) and European Directive 2010/63/EU (47). All work is approved by the University of Aberdeen Animal Welfare and Ethical Review Body (AWERB). A total of four mice were used in this study.

Peritoneal macrophages were isolated from 12- to 16-week-old female mice. Mice were euthanized by cervical dislocation and injected intraperitoneally with PBS containing 5 mM EDTA, and the resident peritoneal cells were harvested. Peritoneal cells were counted and seeded into 12-well plates at a density of 5 × 10^5^ cells/well in RPMI 1640 supplemented with 10% heat-inactivated FBS, 100 U/ml penicillin, and 100 mg/ml streptomycin. Cells were incubated at 37°C in a humidified atmosphere containing 5% CO_2_ overnight. The next day, nonadherent cells were removed by gently washing them with prewarmed PBS. Peritoneal macrophages were either left untreated or activated with 100 ng/ml IFN-γ/LPS for 4 h prior to coincubation with *C. albicans*.

### Flow cytometry analysis.

Macrophages were detached by incubation with ice-cold 5 mM EDTA in PBS for 15 min. Cells were washed once with FACS buffer (1% [wt/vol] BSA–0.5 mM EDTA in PBS), followed by an Fc receptor blockade with human Fc Block (BD Biosciences) in FACS buffer for 10 min at room temperature. Cells were stained for surface antigens with fluorochrome-conjugated (Brilliant Violet 421, FITC, phycoerythrin [PE]-Cy7, peridinin chlorophyll protein [PerCP]-Cy5.5, antigen-presenting cells [APC]) antibodies against human CD14 (MφP9), CD68 (Y1/82A), CD80 (L307.4), CD163 (GHI/61), and CD206 (19.2) (BD Biosciences) for 30 min at room temperature in the dark. Cells were washed once with FACS buffer before being fixed with 4% formaldehyde and analyzed by flow cytometry within 24 h. Samples were acquired using a BD LSRII flow cytometer equipped with BD FACS Diva software (BD Bioscience). FlowJo (TreeStar) was used for the final analysis.

### *C. albicans* caspofungin and thimerosal treatment and chitin extraction.

*C. albicans* strains used in this study are listed in Table 1 and were maintained as glycerol stocks at −80°C. When required, strains were plated onto YPD agar (1% [wt/vol] yeast extract, 2% [wt/vol] peptone, 2% dextrose, and 2% agar) and incubated at 30°C for 48 h. Fungal chitin from *C. albicans* yeast cells was extracted and purified as described previously (12). For *Candida*-macrophage cocultures, yeast cells were precultured twice in 10 ml YPD broth, first for 16 h at 25°C at 200 rpm and then for 24 h at 37°C at 200 rpm. Yeast cells were harvested, washed three times with sterile PBS, counted, and diluted to the desired density. To obtain caspofungin-treated yeast cells that presented chitin on their cell surfaces, yeast cells were inoculated to stationary phase in YPD broth at 30°C at 200 rpm and then diluted in fresh YPD broth (starting optical density at 600 nm [OD_600_] = 0.2) and grown to mid-exponential phase in the presence or absence of caspofungin (3.2 µg/ml). Cells were washed three times with PBS and incubated with 50 mM thimerosal overnight in the dark before being washed repeatedly with PBS and used for stimulation assays or staining for cell wall chitin exposure. Inactivation of *C. albicans* cells by thimerosal was confirmed by plating cells onto YPD agar and incubating them at 30°C for 5 days.

### Phagocytosis assay.

Following coincubation with *C. albicans* for 3 h at 37°C in a humidified atmosphere containing 5% CO_2_, samples were fixed with 4% (wt/vol) paraformaldehyde overnight at 4°C. Cells were washed three times with PBS and blocked with goat serum (1/20) in PBS for 30 min at room temperature. Nonphagocytosed *Candida* cells were stained with a fluorochrome-conjugated rabbit polyclonal anti-*C. albicans* antibody (Acris Antibodies) diluted 1/250 in 1% (wt/vol) BSA in PBS for 1 h at room temperature, followed by three washes with 0.05% (wt/vol) BSA in PBS containing 0.05% (vol/vol) Tween 20 (BSA/PBS-T). Macrophages were permeabilized with 0.5% (vol/vol) Triton X-100 in PBS for 10 min at room temperature, and then samples were washed three times with BSA/PBS-T and incubated with fluorochrome-conjugated phalloidin (1/1,000; Sigma-Aldrich) and 10 µg/ml calcofluor white (CFW) to distinguish nonphagocytosed (FITC- and CFW-positive) from phagocytosed (CFW-positive) fungal cells. Fluorescence was observed with a Zeiss Axio Observer Z1 microscope equipped with a Zeiss MRm digital camera and Zeiss AxioVision software. Images were analyzed with ImageJ. Phagocytic activity was calculated as a phagocytic index (PI) as described before (48), using the following formula: PI = (percentage of phagocytic cells containing ≥1 *Candida* cell) × (mean number of *Candida* cells/phagocytic cell containing *Candida*).

### *Candida* killing assay.

Activated macrophages were coincubated with *C. albicans* for 3 h at 37°C in a humidified atmosphere containing 5% CO_2_. To determine the killing of *C. albicans* by macrophages, the supernatant was removed and macrophages were lysed in 200 µl 0.01% SDS in water to release fungal cells. A serial dilution was performed and plated onto YPD agar. After an incubation period of 24 h at 30°C, numbers of CFU were counted and *Candida* survival/killing was determined by comparing cocultures to *C. albicans* control cultures without macrophages.

### Immunoblots.

Protein expression was analyzed by Western blot analysis. After coincubation, macrophages were washed once with ice-cold PBS before proteins were extracted by lysing cells with 2× Laemmli buffer (49). Samples were heated for 10 min at 95°C and centrifuged for 5 min at 13,000 rpm to pellet cellular debris. Protein samples (20 µl) were separated by SDS-PAGE using the XCell SureLock minicell system (Invitrogen) with NuPAGE Novex Bis-Tris 4 to 12% precast gels (Invitrogen) in NuPAGE MOPS (morpholinepropanesulfonic acid)-SDS running buffer (Invitrogen) as per the manufacturer’s instructions. Proteins were then transferred to Invitrolon polyvinyl difluoride (PVDF) membranes (Invitrogen) in NuPAGE transfer buffer using the XCell II blot module (Invitrogen). Following transfer, the membranes were blocked in 10% (wt/vol) nonfat dry milk in TBS-T (0.1% Tween 20 in TBS) for 1 h at room temperature. Membranes were washed with TBS-T and incubated with primary antibodies in 5% BSA in TBS-T overnight at 4°C, followed by incubation with appropriate horseradish peroxidase (HRP)-conjugated secondary antibodies diluted in 5% BSA–TBS-T for 1 h at room temperature. Arginase-1 was detected using a polyclonal sheep anti-arginase 1 antibody (1/200; R&D Systems), followed by rabbit anti-sheep IgG-HRP (1/1,000; Abcam, Inc.); iNOS was detected using a rabbit polyclonal anti-NOS (pan)antibody (1/500; Cell Signaling) followed by goat anti-rabbit IgG-HRP (1/1,000; Cell Signaling), and β-actin was detected using an HRP-conjugated rabbit monoclonal anti-β-actin antibody (1/1,000; Cell Signaling). Membranes were washed in TBS-T, and signals were detected using the LumiGLO chemiluminescent substrate (Cell Signaling). Signals were quantified using a Fusion FX7/SL chemiluminescence and fluorescence combination imaging system.

### Arginase activity and NO production assays.

Arginase activity was determined using the arginase activity assay kit (Sigma-Aldrich) by following the manufacturer’s instructions. In short, macrophages were washed once with ice-cold PBS before being lysed for 10 min by the addition of 100 µl of 0.4% Triton X-100 in 10 mM Tris-HCl, pH 7.4, supplemented fresh with 1 µM pepstatin A and 1 µM leupeptin. Samples were centrifuged for 10 min at 4°C and 13,000 rpm to remove cellular debris. Lysates were transferred into fresh tubes and stored at −20°C until analyzed. Nitric oxide production was determined using the Griess reagent kit for nitrite determination (Molecular Probes).

### Cell wall staining.

*C. albicans* cells were harvested by washing macrophages with PBS to collect nonphagocytosed fungal cells. Macrophages were then lysed with 0.01% SDS in water to release phagocytosed fungal cells. Fungal cells were washed three times with FACS buffer (1% [wt/vol] BSA–0.5 mM EDTA in PBS) and blocked with 5% goat serum for 30 min at room temperature before being stained with 5 µg/ml purified human Fc–dectin-1 for 1 h on ice. Samples were repeatedly washed before being incubated with fluorochrome-conjugated anti-human IgG Fc antibody (1/250; Life Technologies, Inc.) and fluorochrome-conjugated wheat germ agglutinin (WGA-FITC) (100 µg/ml; Sigma-Aldrich) for 45 min on ice. Cells were washed and stained with CFW (25 µg/ml; Sigma-Aldrich) for 10 min before being intensively washed and finally fixed with 4% (wt/vol) paraformaldehyde. Fluorescence was observed with a Zeiss Imager M2 equipped with a Zeiss MRm digital camera and Zeiss AxioVision software. Images were analyzed with ImageJ.

### Statistical analyses.

All experiments included two biological replicates per donor and were performed at least 3 times, with the exception of the experiments whose results are shown in Fig. 5 and S1 (performed twice), and revealed comparable results. Values are presented as means ± standard deviations (SD) (*n* = the number of blood donors/mouse). Statistical significance was determined using GraphPad Prism 5 (as applicable) using a two-tailed paired Student *t* test and one-way analysis of variance (ANOVA) followed by Tukey *post hoc* analysis. A *P* value of 0.05 or less was considered significant.
