# Supplementary figures and images for: Candida albicans Chitin Increases Arginase-1 Activity in Human Macrophages, with an Impact on Macrophage Antimicrobial Functions
Source: mBio. 2017 Jan 24;8(1):e01820-16. doi: 10.1128/mBio.01820-16 (PMC5263244; doi:10.1128/mBio.01820-16)

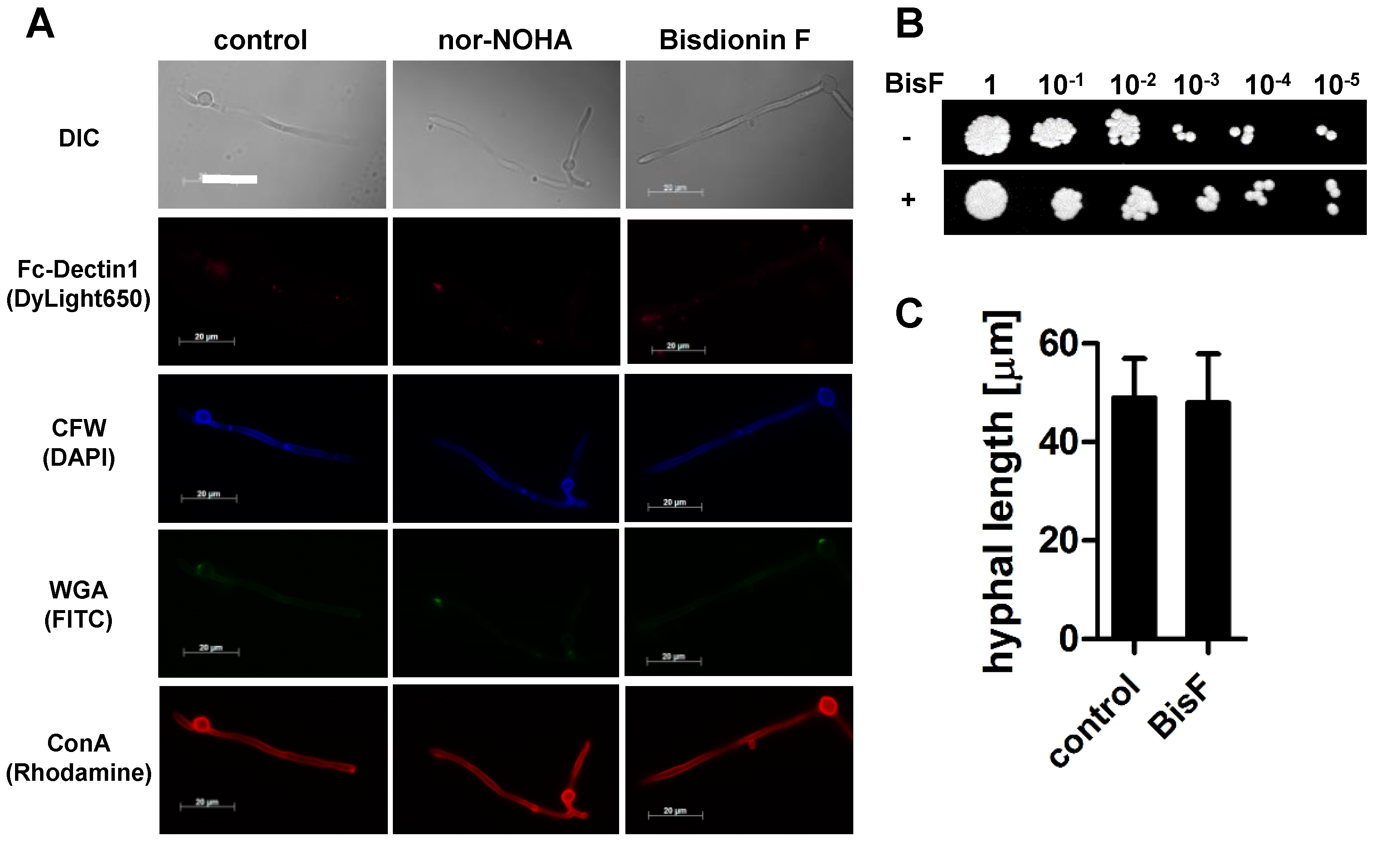

Supplement: FIG S1 [file mbo002173157sf1.tif]
